# {110} Surface‐Exposed Bi_3.15_Nd_0.85_Ti_3_O_12_ Ferroelectric Nanosheet Arrays on Porous Ceramics as Efficient and Recyclable Piezo‐Photocatalysts

**DOI:** 10.1002/smll.202410145

**Published:** 2025-01-27

**Authors:** Yan Zhao, Yan Zhang, Xuefan Zhou, Kaiyu Feng, Qianqian Xu, Hanyu Gong, Di Zhai, Mingyang Yan, Dou Zhang, Chris Bowen

**Affiliations:** ^1^ State Key Laboratory of Powder Metallurgy Central South University Changsha Hunan 410000 China; ^2^ Department of Mechanical Engineering University of Bath Bath BA2 7AY UK

**Keywords:** H_2_ production, ferroelectric polarization, piezo‐photocatalysis, porous ceramics

## Abstract

Bismuth‐layered ferroelectric nanomaterials exhibit great potential for piezo‐photocatalysis. However, a major challenge lies in the difficulty of recovering the catalytic powders, raising concerns regarding secondary pollution of water. In this work, a novel hierarchical porous ferroelectric ceramic containing {110} surface‐exposed Bi_3.15_Nd_0.85_Ti_3_O_12_ (BIT‐Nd) nanosheet arrays is grown on a porous ceramic matrix for efficient and recyclable piezo‐photocatalysis. By controlling the BIT‐Nd loading level of the nanosheets, the piezo‐photocatalytic degradation efficiency of a Rhodamine B(RhB) (*C*_0_ = 10 mg L^−1^) solution reached an optimum value of 97.1% in 100 min with a first‐order kinetic rate constant, *k*, of up to 0.0321 min^−1^ in Bi_3.15_Nd_0.85_Ti_3_O_12_‐20 (BITNd‐20) with a mass ratio of hydrothermal products to ceramics of 20%. In the presence of BITNd‐20, a surprising H_2_ yield rate of 130 µmol·h^−1^ is achieved without using any cocatalyst or scavenger. Specially, the beneficial role of snowflake structures on piezoelectric potential amplification and introducing nanosheets with exposed {110} surfaces on hydrogen evolution reaction (HER) activity, piezoelectric potential output, and catalytic performance of porous ceramics has been revealed. This unique design strategy provides a new approach to enhance the piezo‐photocatalytic activity by addressing environmental issues and enhancing catalytic performance to yield cleaner energy.

## Graphical abstract

This study introduces a hierarchical design strategy for creating nanosheet‐modified porous piezoelectric ceramics. By incorporating nanosheets with exposed {110} surfaces, the HER activity, piezoelectric response, and photoelectric response of the porous ceramics are enhanced. This results in high efficiency, stability, and durability in piezo‐photocatalysis, while also overcoming secondary pollution issues of water associated with the complex recovery of powder‐based catalysts.

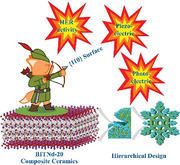

## 1 Introduction

The global energy crisis and environmental pollution are becoming increasingly serious, posing a significant challenge to economic development and social stability.^[^ ^1^ ^]^ Therefore, the degradation of pollutants in water and the development of H_2_ due to its high energy density have become a key challenge to overcome.^[^ ^2^ ^]^ Hydrogen (H₂) generation is considered to be a promising and abundant renewable fuel of high energy density, making it a key solution for addressing the global energy crisis and reducing environmental pollution.^[^ ^3^ ^]^ Recently, piezocatalysis has been exploited as a new approach that exploits the spontaneous polarization of ferroelectric materials, and has gained attention for its potential to enhance hydrogen production.^[^ ^4^ ^]^ Photocatalytic technologies, which are able to convert solar energy to chemical energy for water splitting and wastewater treatment, have shown significant potential due to their low cost and environmental benefits.^[^ ^4c^ ^]^ However, the practical application of photocatalysis is limited due to challenges associated with a high combination rate of electron‐hole pairs, low solar energy utilization and difficulties in the recovery of the photocatalyst, which can lead to secondary pollution.^[^ ^5^ ^]^ In order to improve the photocatalytic efficiency, much effort has been made in the area of defect engineering,^[^ ^6^ ^]^ heterojunction structure construction,^[^ ^7^ ^]^ and morphology maneuvering.^[^ ^8^ ^]^ In particular, the piezoelectric photoelectron effect, which couples both piezoelectric and photoelectric effects, has been proposed by Wang et al.^[^ ^9^ ^]^ The built‐in electric field generated by piezoelectric materials when subject to an external mechanical load can be used to facilitate electron‐hole pair separation, and modulate the energy band structure to promote the reduction‐oxidation reaction.^[^ ^10^ ^]^ It is worth noting that this coupling mechanism collects not only solar energy but also external vibrational and mechanical energy, which can be considered to be another renewable and low‐cost energy source,^[^ ^2a^ ^]^ thereby providing a sustainable approach for both energy production and environmental remediation.^[^ ^4c^ ^]^

In recent years, the coupling of piezoelectric and photovoltaic effects in piezoelectric semiconductor nanomaterials has been extensively investigated, with the material system extending from metal oxide ZnO^[^ ^11^ ^]^ and perovskite structure ferroelectrics BaTiO_3_^[^ ^12^ ^]^ to layered 2D materials MoS_2_^[^ ^13^ ^]^ and g‐C_3_N_4_.^[^ ^14^ ^]^ Among the piezo‐phototronic semiconductors, Bi_4_Ti_3_O_12_ (BIT) with its layered structure has attracted increasing attention. The alternating stacking of [Bi_2_O_2_]^2+^ bismuth oxide layers and [Bi_2_Ti_3_O_10_]^2−^ perovskite layers along the *c*‐axis results in the BIT crystals growing in a 2D shape, which is easily deformed when subjected to mechanical vibrations.^[^ ^15^ ^]^ In addition, the unique layered structure makes the electron migration characteristics in the BIT system exhibit show anisotropy along the in‐plane *a(b)*‐axis and along the out‐of‐plane *c*‐axis direction.^[^ ^16^ ^]^ However, the rate of migration of photogenerated electrons along the *c*‐axis is slower than the *a(b)* axis due to the large interlayer potential barrier, and this leads to an increase in the probability of photogenerated hole and electron combination.^[^ ^17^ ^]^ Consequently, during the migration of charge carriers from the bulk of the BIT‐based ceramic to the {110} surface along the *a*(*b*)‐plane, the probability of charge carriers recombining is lower than other surfaces. Moreover, the spontaneous polarization of BIT along the *a*‐axis and along the *c*‐axis is 40 and 4 µC cm^−2^ respectively.^[^ ^18^ ^]^ Hence, reducing the thickness in the *c*‐direction and increasing the area of the {110} surface are both effective methods to minimize the electron‐hole combination, while simultaneously enhancing the ferroelectric response, leading to the high piezoelectric potential to improve the piezo‐photocatalytic performance of BIT‐based catalysts. Metal ion doping is also a popular and effective strategy to modulate the piezoelectric and photoelectric properties of BIT‐based crystals. First, the doping of BIT with rare earth metal ions effectively enhances visible light absorption and suppresses the combination of electron‐hole pairs.^[^ ^19^ ^]^ Second, replacing Bi^3+^ with smaller lanthanide ions such as Nd^3+^ significantly enhanced the spontaneous polarization.^[^ ^20^ ^]^ In our recent work, the reduction in bandgap and increase in polarization field achieved through doping BIT with Nd have led to the remarkable activity of Bi_3.15_Nd_0.85_Ti_3_O_12_ nanosheets for hydrogen production and dye degradation.^[^ ^21^ ^]^

Despite the remarkable achievements of piezoelectric semiconductor nanomaterials, the significant challenge of the secondary pollution of water due to the difficulty in powder recovery continues to persist, which hinders the widespread application of powder‐based piezo‐photocatalysis.^[^ ^22^ ^]^ It is therefore of significance to convert these materials from a powder form to a bulk form. The preparation of powder catalysts in composite^[^ ^23^ ^]^ and porous ceramics^[^ ^24^ ^]^ forms can effectively alleviate the problem of difficult recycling of powder catalysts. Feng et al.^[^ ^23a^ ^]^ reported on the formation of a Bi_4_Ti_3_O_12_@PDA/PVDF composite foam, which was able to degrade an indigo carmine (IC) dye by ≈96.1% within 40 min, when subject to ultrasonic vibrations. The contact area between the piezoelectric phase and the dye in 0–3 type piezoelectric composite is limited, due to the isolated nature of the filler particles, which reduces the degradation efficiency of the embedded, or semi‐embedded, bulk catalyst. To increase the direct contact area between the piezoelectric ceramic and the solution, the introduction of pores in the material is an economical and effective approach. In this regard, Guar et al.^[^ ^24b^ ^]^ introduced random pores into BaTiO_3_ ceramics by adding 30 wt. % of a pore‐forming agent. The piezoelectric catalytic degradation activity of randomly distributed porous BaTiO_3_ ceramics increased by ≈60% compared with dense samples, with a *k* value of 0.0080 min^−1^. The disadvantage of introducing pores via this technique is that there are closed pores within the microstructure, which greatly reduces the exchange rate between the ceramic and the water/solution flow.

Compared with ceramics with randomly distributed porosity, the introduction of aligned highly connected pore structures in ceramics can effectively improve the contact interface between flowing water and the ferroelectric ceramic, which makes the internal utilization of the ceramics higher and significantly increases the degradation rate.^[^ ^25^ ^]^ In our recent work, we have demonstrated that by introducing aligned interconnected channels in Ba_0.75_Sr_0.25_TiO_3_ ceramics, the piezoelectric catalytic water splitting efficiency increases by 253% compared to the dense.^[^ ^2b^ ^]^ However, the effects of the porosity and structure of the pore channels on the piezo‐photocatalytic capability are yet to be demonstrated, which exhibits high potential to further improve their catalytic activity to realize their practical applications. In order to produce porous ceramics for wastewater purification and hydrogen generation from water decomposition, developing recyclable porous ceramics with aligned pores and modification of its pore channels within the ceramic body is an effective approach to solve the challenge of secondary contamination associated with powders and enhancing piezo‐photocatalytic performance.

Therefore, in this work, Bi_3.15_Nd_0.85_Ti_3_O_12_ (BIT‐Nd) micro‐meter scale powders were used to prepare an aligned porous ceramic matrix in the shape of multi‐edge snowflakes by freeze‐casting. Nanosheets exposing the {110} surface were grown on the pore channels of the porous ceramics matrix by a hydrothermal method to form BITNd‐*x* (*x* = 0, 10, 20, 30 wt.%) porous composite ceramics. Specifically, these nanosheets are oriented along the *a(b)*‐axis, effectively increasing the area of the {110} surface. Ferroelectric ceramics with aligned pores were demonstrated for the first time for piezo‐photocatalytic degradation of RhB and water decomposition. This piezo‐photocatalyst with porous structure and complex pore channel structure is shown to exhibit good shape stability and ease of dimensional tunability, which meets the requirements of recyclability and high efficiency in the catalytic process. This new form of porous composite offers a promising prospect to provide materials with enhanced piezo‐photocatalytic performance in an attempt to tackle the increasing global water pollution and energy crisis.

## 2 Results and Discussion

### 2.1 Materials Characterization

**Figure 1:**
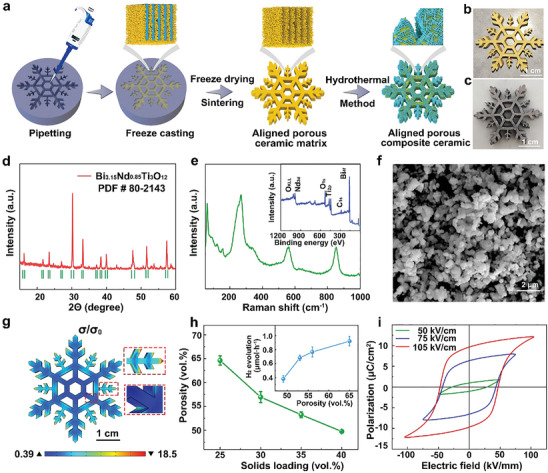
Morphology and microstructure characterization of the BIT‐Nd powders and porous ceramics. a) Schematic of the fabrication of BIT‐Nd porous composite ceramic. Photographs of b) BIT‐Nd porous ceramic matrix and c) BIT‐Nd porous composite ceramic. d) XRD patterns, e) Raman spectra with XPS spectra and f) SEM images of the BIT‐Nd powders. g) Stress distribution in a simulated snowflake using COMSOL simulations. h) Relationship between ceramic solid loading, porosity and H_2_ evolution rate. i) Ferroelectric hysteresis loop of BIT‐Nd porous ceramic matrix with a porosity of 64.6 vol.% at various applied electric fields.

The synthesis process of the BITNd‐*x* (*x* = 0, 10, 20, 30 wt.%) porous composite ceramic is shown in **Figure** **1**a. First, a BIT‐Nd ceramic micro‐metre scale powder‐water suspension was poured into a silica gel mould, and the suspension was frozen in a liquid nitrogen container. The large degree of supercooling led to ice crystals growing anisotropically and forming pore channels. After the sublimation of the ice crystals, a ceramic was formed with an orientated and porous structure. After sintering, a hydrothermal method was carried out to load nanosheets onto the surface of the pore channels of the porous ceramics to form BITNd‐*x* (*x* = 0, 10, 20, 30 wt.%) porous composite ceramics. Images of the BIT‐Nd porous ceramic matrix and BIT‐Nd porous composite ceramic are shown in Figure 1b,c. XRD patterns of the BIT‐Nd powders in the range of 10°–60° are shown in Figure 1d, which exhibited a stable solid solution after the introduction of Nd into the BIT structure. Figure 1e shows the Raman and XPS spectra of BIT‐Nd at room temperature with the main characteristic peaks of 60, 90, 122, 158, 260, 540 and 840 cm^−1^; the characteristic peaks were similar to those of other bismuth layer‐structured ferroelectrics.^[^ ^26^ ^]^ The vibrations at different frequencies were closely related to the atomic mass, where vibrations in the low‐frequency mode were mainly due to the motion of the heavy Bi^3+^, while the [TiO_6_]^2−^ octahedron vibrations matched the appearance of characteristic peaks at ∼260, ∼540, and ∼840 cm^−1^. Notably, the Raman characteristic peaks were broader, which was due to the orthogonal distortion of the [TiO_6_]^2−^ octahedra as a result of the formation of chemical bonds after doping.^[^ ^27^ ^]^ The XPS spectra of the BIT‐Nd powders indicated the presence of the elements Bi, Ti, O and Nd in the powder, where the detected signal of the carbon element was a result of adsorbed carbon contamination of the sample. Figure 1f and Figure S1 (Supporting Information) show an SEM image and particle size distribution of BIT‐Nd powders, which exhibited irregular shapes and powder sizes ranging from 0.25–2.75 µm with an average size *D_v_(50)* of 0.75 µm.

The stress distributions of piezoelectric ceramics formed into a variety of shapes and subject to ultrasonic treatment were simulated using the COMSOL multiphysics field simulation method, as shown in Figure 1g and Figure S2 (Supporting Information). When the material was subjected to a force, the stress was primarily concentrated at the edges and sharp corners of the ceramic. The maximum stress observed in the snowflake structure was 18.5 times greater than the input pressure, which was significantly larger than conventional rectangular, hexagonal, and less‐legged snowflake structures under the same level of input pressure. There is a linear relationship between the stress and the piezoelectric potential output *V* according to Equations (1) and (2): ^[^ ^28^ ^]^

(1) D=dT+εE

(2) $$V=\frac{DH}{\varepsilon }\;$$

where *D* is electric displacement, *d* is the piezoelectric charge coefficient, *ε* is permittivity, *E* is the electric field and *H* is the thickness of the piezoelectric. It can be clearly seen that when the stress level is increased, the change in potential will increase, causing a further increase in the piezoelectric potential output. Figure S2 (Supporting Information) shows the piezoelectric potential output of different structures, where the snowflake structured piezoelectric ceramic exhibits a greater piezoelectric potential output, indicating that it is more promising for piezoelectric catalysis.

The relationship between solid loading, porosity and hydrogen evolution rate is shown in Figure 1h. The solid loading of the suspensions for freeze casting were 25, 30, 35, and 40 vol.%, corresponding to the porosities of 64.6, 57.0, 53.3, and 49.7 vol.%, respectively. With an increase in porosity fraction, the hydrogen evolution rate increased from 0.38 to 0.92 µmol^−1^·h^−1^. This enhancement was primarily attributed to a relatively high effective diffusion coefficient resulting from the widening of the parallel pores from 5 to 15 µm, as shown in Figure S3 (Supporting Information). Therefore, snowflake‐type BIT‐Nd aligned porous ceramics with a solid loading of 25 vol.% and a porosity of 64.6 vol.% were used in subsequent experiments. Figure 1i shows the polarisation‐electric field (*P*‐*E)* loops of aligned porous BIT‐Nd ceramics with a porosity of 64.6 vol.% subject to different applied electric fields that range from 50 to 105 kV cm^−1^. The remanent polarization (*P_r_*) of the ceramics was ≈10 µC cm^−2^ at an applied electric field of 105 kV cm^−1^, which exhibited well‐saturated *P*‐*E* switching curves.

**Figure 2:**
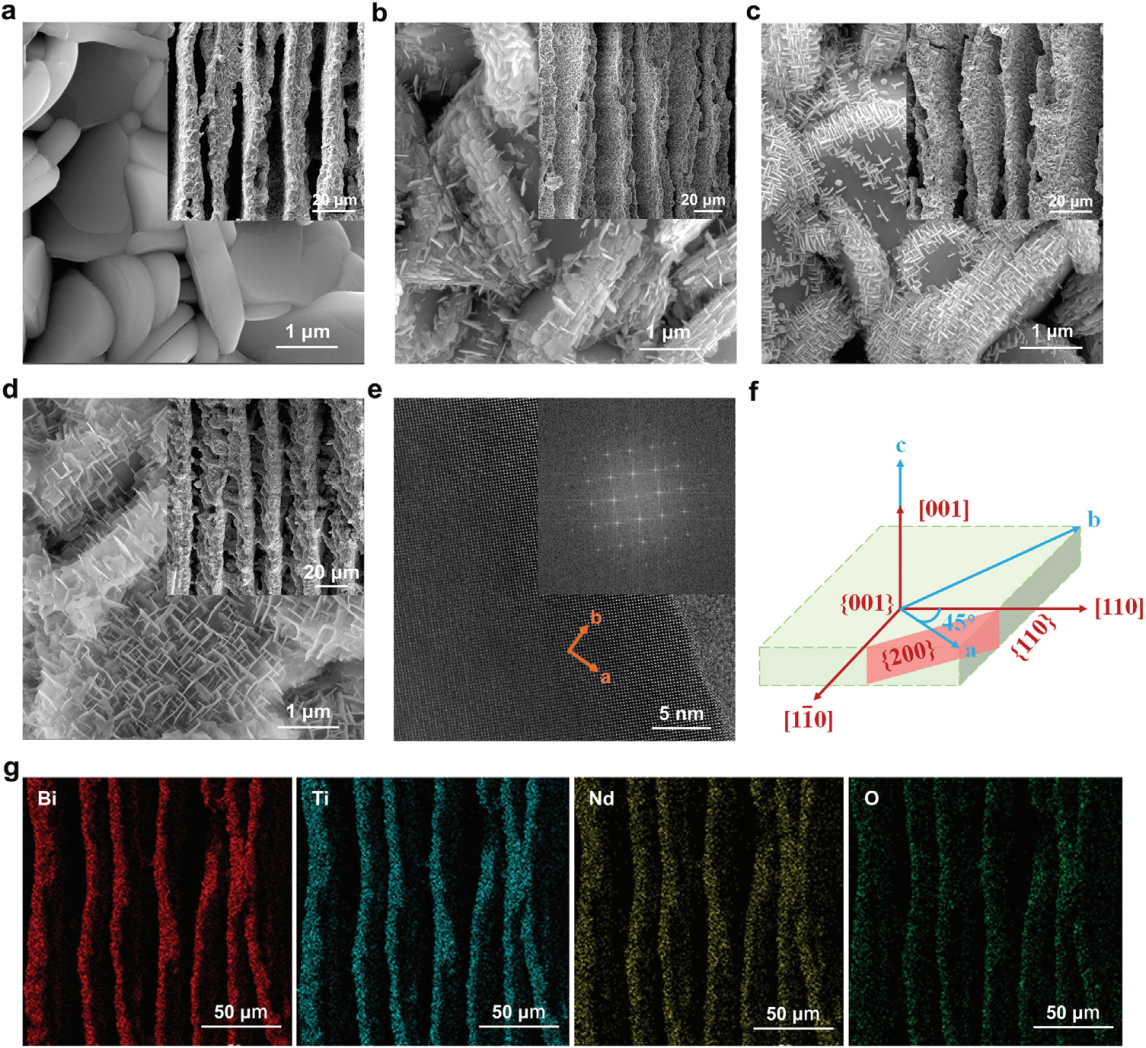
SEM images of aligned porous BITNd‐*x* composite ceramics: a) *x* = 0 wt.%; b) *x* = 10 wt.%, c) *x* = 20 wt.%; d) *x* = 30 wt.%. e) High‐resolution TEM image with the Fast Fourier transform (FFT) pattern of the surface‐loaded nanosheet of BITNd‐*x* porous composite ceramic. f) Schematic of the crystal orientation of the nanosheet. g) Mapping images of BITNd‐30 porous composite ceramic.

**Figure** **2**a–d shows the SEM images of BIT‐Nd porous composite ceramic (BITNd‐*x*, *x* = 0, 10, 20, 30) at low and high magnification. At low magnification, the lamellar structure of all composite ceramics can be clearly observed, which was attributed to the directional growth of ice crystals during the freeze casting process resulting in the ceramic particles being pushed between the directionally grown ice crystals.

As shown in Figure 2a, due to the anisotropy of the grain growth process in the bismuth titanate‐based system, the particles of BITNd‐0 composite ceramics were smooth and disc‐shaped. The anisotropy of growth was closely related to the crystal structure and the bismuth titanate‐based ceramics had alternating rows of bismuth‐oxygen and pseudo‐calcite layers in the *c*‐direction, which were connected by weak bonds formed by O^2−^.^[^ ^29^ ^]^ Therefore, this unique structure led to a much higher growth rate of the grains along the *a(b)*‐axis than the growth rate along the *c*‐axis. As shown in Figure 2b–d, the loading of nanosheets was regulated by controlling the mass of the reactants during the hydrothermal process. The degree of loading exhibited a positive correlation until it reached 30 wt.% of the mass of the substrate, achieving complete coverage of nanosheets in the ceramic matrix.

Figure 2e shows the TEM images of the surface‐loaded nanosheet of BITNd‐*x* porous composite ceramic, which shows clear and uniform lattice fringes. In addition, the Fast Fourier transform (FFT) image illustrates that the nanosheets exhibit a single crystal characteristic. Based on these results, the crystal structure and orientation schematic are developed, as shown in Figure 2f, where the exposed surface of the nanosheets represents the {110} surface. The growth process of nanosheets can be observed to occur in two distinct steps: initially, nanosheets oriented along the <110> direction grow on the lateral {110} surface of the substrate particles. Subsequently, nanosheets with a <110> direction continued to grow on the basal {001} surface, until the surface of the ceramic particles was fully covered by the nanosheets. At this point, the presence of nanosheets increases the area of the {110} surface in BITNd‐*x* porous composite ceramics, with their arrangement exposing it prominently. As shown in Figure 2g, elements Bi, Ti, Nd and O were uniformly distributed in the BITNd‐30 porous composite ceramic, indicating that Nd is successfully doped with BIT.

### 2.2 Catalytic Activity for Dye Degradation

**Figure 3:**
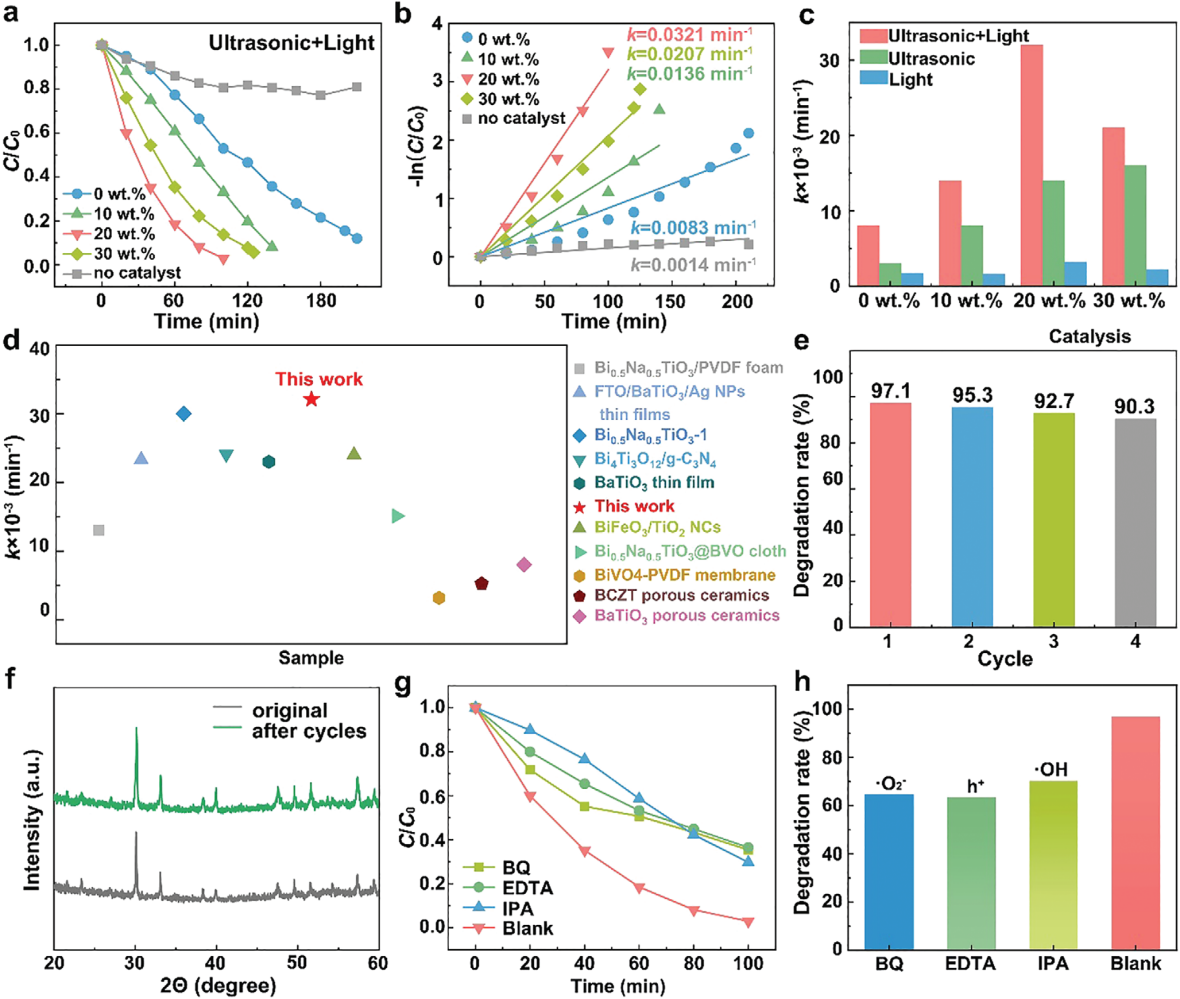
Characterization of piezoelectric‐photocatalytic properties of BITNd‐*x* (*x* = 0, 10, 20, 30) composite ceramics. a) Concentration variation – time curves and b) first‐order kinetic rate constant of BITNd‐*x* composite ceramics piezo‐photocatalytic degradation of RhB (*C*_0_ = 10 mg L^−1^). c) Comparison of the *k* values of BITNd‐*x* composite ceramics for the degradation of RhB solution (*C*_0_ = 10mg L^−1^) under different conditions. d) Comparison of *k* value with previously reported work. e) Cycle test and f) XRD patterns of BITNd‐20 composite ceramics during four piezo‐photocatalytic cycles. Change of g) concentration over time, and h) degradation rate of RhB by piezo‐photocatalytic degradation of BITNd‐20 composite ceramics after addition of different free radical scavenger BQ, EDTA and IPA.

The influence of the nanosheet density of the composite ceramics on piezo‐photo catalytic properties was evaluated. In **Figure** **3**a, the piezo‐photocatalytic degradation of RhB (*C*_0_ = 10 mg L^−1^) via the BITNd‐*x* composite ceramics is shown, with the duration of each experiment determined by the RhB degradation rate reaching 90%. As a control, an aqueous solution of RhB (*C*_0_ = 10 mg L^−1^) without any catalyst was also reacted when subjected to ultrasound and light for 210 min, showing negligible auto‐degradation efficiency. Figure 3b shows the corresponding first‐order kinetic rate constants *k* values of 0.0083, 0.0136, 0.0321, and 0.0207 min^−1^ for BITNd‐0, BITNd‐10, BITNd‐20, and BITNd‐30 composite ceramics, respectively, under ultrasound and light conditions. This indicates that the piezo‐photocatalytic performance of BITNd‐*x* composite ceramics increases as the nanosheet density increases until it reaches 20 wt.%, where the *k* reaches a maximum value of 0.0321 min^−1^. However, with a further increase in nanosheet density, the photocurrent density decreases due to an increase in the electron‐hole recombination rate, as shown in Figure S4 (Supporting Information), which in turn reduces the piezo‐photocatalytic activity.

The rate constants, *k*, for photocatalysis, piezocatalysis, and piezo‐photocatalysis of BITNd‐*x* composite ceramics are summarised in **Table** **1** and are shown in Figure 3c. In addition, the corresponding C/*C*_0_ ‐t plots and the fitting of kinetic curves are shown in Figure S5 (Supporting Information); where *C* is concentration, *C*_0_ is initial concentration and *t* is time. The results confirmed that the photocatalytic activity of the BITNd‐ *x* composite ceramics is better than that of the porous ceramics which are not loaded with nanosheets, reaching a maximum at 20 wt.%, which was consistent with the transient photocurrent response shown in Figure S4 (Supporting Information). The Apparent Quantum Yield (AQY) values at a wavelength of 350 nm for the BITNd‐ *x* composite ceramics are displayed in Figure S6 (Supporting Information), with BITNd‐20 also exhibiting the highest AQY. The photocatalytic activity of BITNd‐20 was compared with the materials reported in the literature,^[^ ^4^, ^30^ ^]^ as shown in Figure S7 (Supporting Information), where the efficiency of BITNd‐20 is superior due to its optimized porous structure and the presence of nanosheets. Figure 3c and Figure S5 (Supporting Information) demonstrate the piezocatalytic process and catalytic activity of BITNd‐*x* composite ceramics when subject to ultrasound in the dark to investigate the effect of the nanosheets on piezocatalytic performance. As shown in Figure 3c, the *k* values of BITNd‐0, BITNd‐10, BITNd‐20, BITNd‐30 were 0.0028, 0.0081, 0.0140, and 0.0162 min^−1^, respectively.

**Table 1:** First‐order kinetic rate constant (*k*) for the photocatalytic, piezocatalytic and photo‐piezocatalytic activity of BITNd‐*x* composite ceramics.

Activity	first‐order kinetic rate constant	BITNd – 0	BITNd – 10	BITNd – 20	BITNd – 30
Photo	*k* × 10^3^ min^−1^	1.7	1.6	3.2	2.2
Piezo	*k* × 10^3^ min^−1^	2.8	8.1	14.0	16.2
Photo‐piezo	*k* × 10^3^ min^−1^	8.3	13.6	32.1	20.7

Of particular note is that the increase in piezocatalytic activity of the BITNd‐*x* composite ceramics was not significant when its loading was increased from 20 to 30 wt.%. This observation was attributed to the rapid increase in active sites accelerating the catalytic reaction when the concentration of RhB was high. However, at the late stage of catalysis, the RhB concentration decreased, resulting in some of the reactive active sites of the catalyst not being utilized, and saturation of the piezocatalytic activity occurred. Furthermore, a remarkable improvement was observed in the combined photo‐piezocatalysis process for all samples compared to either piezoelectric catalysis alone or photocatalysis alone. Specifically, the *k* value of combined piezo‐photocatalysis exceeded the sum of photocatalysis and piezocatalysis alone. This large improvement suggests that the polarization‐modulated built‐in electric field in the BITNd‐*x* composite ceramics facilitates the separation and transfer of carriers, thereby achieving a strong level of coupling between piezoelectric and photoexcitation in the BITNd‐*x* composite ceramics.

The piezo‐photocatalytic first order rate constant, *k*, values of the BITNd‐20 composite ceramics were compared with those of the recently reported bulk catalysts, as shown in Figure 3d. Among the various piezo‐photocatalyst systems investigated, the BITNd‐20 composite ceramics exhibited outstanding catalytic performance, which is highly promising and valuable for practical applications. In order to assess the long‐term operation stability and durability of the catalysts, multiple cycling experiments were necessary. Figure 3e shows the degradation rate changes of BITNd‐20 in four cycling experiments, revealing that the degradation rate of RhB remained above 90% even during the fourth cycling experiment of BITNd‐20 composite ceramics. The slight decrease in dye degradation efficiency that is observed is due to the reduced adsorption of RhB dye on the catalyst surface after repeated cycles, as shown in Figure S8 (Supporting Information). Furthermore, the BITNd‐20 exhibits superior cycling stability over four cycles compared to other bulk materials, as shown in Figure S9 (Supporting Information).^[^ ^23^, ^30^, ^31^ ^]^ Moreover, the unchanged XRD spectra before and after cycling also demonstrated its high durability and stability, as shown in Figure 3f. In addition, SEM images of BITNd‐20 before and after the catalytic process (Figure S10, Supporting Information) confirm that the catalysts retain their structural integrity, thereby providing deeper insights into their reliability.

In order to investigate the mechanism of piezo‐photo catalysis more clearly, benzoquinone (BQ), ethylenediaminetetraacetic acid disodium (EDTA), and isopropyl alcohol (IPA) were intentionally added as the scavengers of superoxide anion (•O_2_^−^), hole (h^+^), and hydroxyl radical (•OH), respectively, for the radical trapping experiments. Figure 3g,h shows that the addition of all three radical scavengers, BQ, EDTA and IPA, had a certain weakening effect on the piezo‐photocatalytic efficiency of the BITNd‐20 composite ceramics, with the degradation rate decreasing from 97.1% (without additives) to 64.6%, 63.6%, and 70.2%, respectively. Among these, it is observed that the addition of EDTA had the most serious effect on the catalytic activity, indicating that h^+^ played the most dominant role in the piezo‐photocatalytic process, and •O_2_^−^ and •OH were important in the piezo‐photocatalysis. The relative amounts of •O₂⁻ and •OH produced under different conditions were determined by EPR measurements, see Figure S11 (Supporting Information). The strongest signals for •O_2_^−^ and •OH were detected during the piezo‐photocatalytic process, indicating that a relatively higher amount of •O_2_^−^ and •OH was produced under this state, compared to ultrasound alone and light alone. Therefore, the possible piezo‐photocatalytic degradation mechanism of RhB is shown in Equations ((3), (4), (5), (6)).

(3) $$\begin{array}{ccc} BITNd & - & x\;composite\;ceramic\overset{\mathrm{light}+\mathrm{ultrasound}}{\to }BITNd\\ & & -x\;composite\;ceramic+h^{-}+e^{-} \end{array}$$

(4) $$O_{2}+e^{-}\to \cdot O_{2}^{-}$$

(5) $$OH^{-}+h^{+}\to \mathrm{OH}$$

(6) $$\cdot \mathrm{OH}+\cdot O_{2}^{-}+h^{+}+dye\;molecules\to CO_{2}+H_{2}O$$

### 2.3 Catalytic Activity for H_2_ Evolution and Mechanism of Piezo‐Photocatalysis

**Figure 4:**
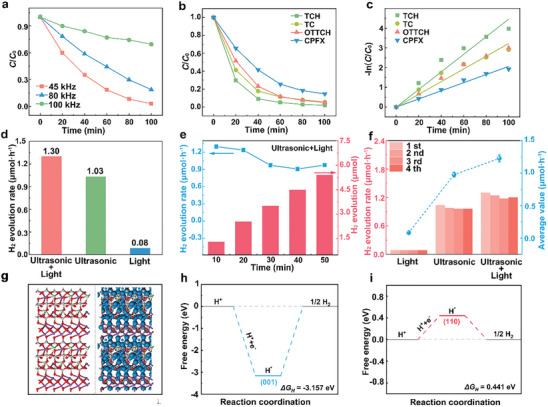
Other catalytic applications of BITNd‐20 composite ceramics: a) Comparison of RhB degradation efficiencies under ultrasound and light at different frequencies. b) Piezo‐photocatalytic degradation efficiencies of pollutants OTTCH, CPFX, TCH and TC and c) kinetic curve fitting. d) H_2_ production rates for photocatalysis, piezocatalysis and piezo‐photocatalysis. e) Amount of H_2_ over time during the piezo‐photocatalysis process. f) Cycling experiments for H_2_ production. g) Atomic structure diagrams and isosurface total electron density distribution of atoms of (110) crystal plane in BIT‐Nd nanosheets (the atoms in red, purple, gray and cyan represent O, Bi, Ti and Nd, respectively. The blue areas represent the total electron density distribution at the isosurface value of 0.5 eÅ^−3^.). Reaction free energy diagram of H_2_ production on h) (001) crystal plane and i) (110) crystal plane.

As shown in **Figure** **4**a, the BITNd‐20 composite ceramics exhibited different piezo‐photocatalytic activities at ultrasound frequencies of 45, 80 and 100 kHz, where the degradation efficiencies of RhB within 100 min were 97.0%, 81.3%, and 31.5%, respectively. This variation can be attributed to the different frequencies of ultrasound affecting the duration of the acoustic cycle. ^[^ ^32^ ^]^ At lower ultrasound frequencies, the longer duration of the acoustic cycle resulted in the formation of sufficiently large cavitation bubbles that underwent violent collapse.^[^ ^33^ ^]^ This diminished chemical effects, such as sonochemical effects, while enhancing mechanical effects such as shock waves, micro‐jetting, and asymmetric collapse, thereby inducing strong localized piezoelectric responses.^[^ ^34^ ^]^ As shown in Figure S12 (Supporting Information), under the application of ultrasound and light, the peak values of characteristic peaks for the four colorless pollutants, tetracycline hydrochloride (TCH), tetracycline (TC), oxytetracycline hydrochloride (OTTCH), and ciprofloxacin (CPFX), decreased over time. To quantify the degradation efficiency, the concentration changes of the four colorless pollutants over time are shown in Figure 4b,c. Within 100 min, the degradation rates of TCH, TC, OTTCH, and CPFX reached 85.4%, 94.5%, 95.0%, and 98.1%, respectively. The corresponding first‐order kinetic constants, *k*, were 0.0446, 0.0323, 0.0322, and 0.0206 min^−1^. This outstanding piezo‐photocatalytic performance further confirmed the universality and potential of BITNd‐20 composite ceramics in catalytic applications.

The piezo‐photocatalytic activity of BITNd‐*x* composite ceramics has been demonstrated to favor the degradation of RhB and colorless pollutants. To investigate the effective role of BITNd‐*x* composite ceramics in HER, hydrogen production experiments under ultrasonic plus light conditions were performed. Figure 4d shows the properties of photocatalytic, piezocatalytic and piezo‐photocatalytic H_2_ production from water splitting using BITNd‐20 composite ceramics, with H_2_ production rates of 0.08, 1.03, and 1.30 µmol·h^−1^, respectively. The H_2_ production rate of BITNd‐20 composite ceramics under combined ultrasound and light was greater than the sum of light alone or ultrasound alone. Such a significant coupling enhancement indicates that the built‐in electric field of the BITNd‐*x* composite ceramics provided the separation and migration of the electron‐hole (e^−^‐h^+^) pairs as a driving force to improve the photocatalytic performance,^[^ ^33^ ^]^ which offers the potential to broaden the application of piezoelectric ceramics in catalysis. Figure 4e shows that under the conditions of ultrasound and light, H_2_ generated by BIT‐Nd steadily increases within 50 min. To evaluate the potential reusability of BITNd‐20 composite ceramics, H_2_ production experiments were repeated four times using recycled materials during photocatalytic, piezocatalytic, and piezo‐photocatalytic processes, as shown in Figure 4f. Similar photocatalytic, piezocatalytic, and piezo‐photocatalytic activities were observed in these consecutive cycles, with average H_2_ production rates of 0.08, 0.98, and 1.23 µmol·h^−1^, respectively, further confirming the stability of the BITNd‐20 composite ceramics.

The significant difference in dye degradation and hydrogen production rates before and after nanosheet modification of BIT‐Nd porous ceramics inspired us to explore the differences in electron density distribution and catalytic activity between the (110) and (001) surfaces. Figure 4g and Figure S13 (Supporting Information) show the atomic structure diagrams and the total electron density distribution of the atomic equivalent surfaces of the (110) and (001) crystal faces of the BIT‐Nd nanosheets, respectively. The calculated electron density distributions reveal that the electron distribution of Bi atoms (depicted as purple atoms) was absent in the (110) and (001) crystal planes at a total electron density distribution at the isosurface value of 0.5 eÅ^−3^. However, the electron distribution was observed near the dopant Nd atoms in both the (110) and (001) crystal planes. This indicates that the presence of Nd enables the (110) and (001) crystal planes of the nanosheet to generate more electrons, facilitating their participation in the catalytic reaction and thereby promoting the catalytic activity.

Density Functional Theory (DFT) calculations were used to evaluate the thermodynamics of hydrogen adsorption and desorption on the (110) and (001) surfaces. There were three step states: the initial state G(H^+^+e^−^), the intermediate state G(H*), and the final state G($\frac{1}{2}$ H_2_). At a pH = 0, the free energy change for hydrogen desorption equals the absolute value of the free energy change for hydrogen adsorption.^[^ ^35^ ^]^ In such an instance, the free energy of hydrogen adsorption (Δ*G_H_*) was typically utilized to characterize the hydrogen evolution reaction (HER) activity, as determined by the following Equation (7):^[^ ^36^ ^]^

(7) $$\Delta \;G_{H}=\Delta E_{ads}+\Delta E_{ZPE}-T\Delta S+\Delta G\left(pH\right)$$

where Δ*E_ads_*, Δ*E_ZPE_*, T, Δ*S* and Δ*G*(*pH*) are the binding energy, the change in zero point energy (ZPE), temperature, the change in entropy and the pH correction term, respectively.^[^ ^37^ ^]^ If Δ*G_H_* is either very negative or very positive, the adsorption will be either too strong or too weak. Therefore, the optimal value of Δ*G_H_* is 0, indicating that the surface binding with hydrogen is neither too weak nor too strong for optimal hydrogen production activity.^[^ ^38^ ^]^ As shown in Figure 4h,i, the Δ*G_H_* values for HER with exposure surfaces (110) and (001) were 0.441 and −3.157 eV, respectively. Since a smaller |Δ*G_H_* | indicated a better HER activity, the stronger hydrogen production catalytic activity of BIT‐Nd nanosheets on the (110) surface compared to the (001) surface can be attributed to the superior Δ*G_H_*.

**Figure 5:**
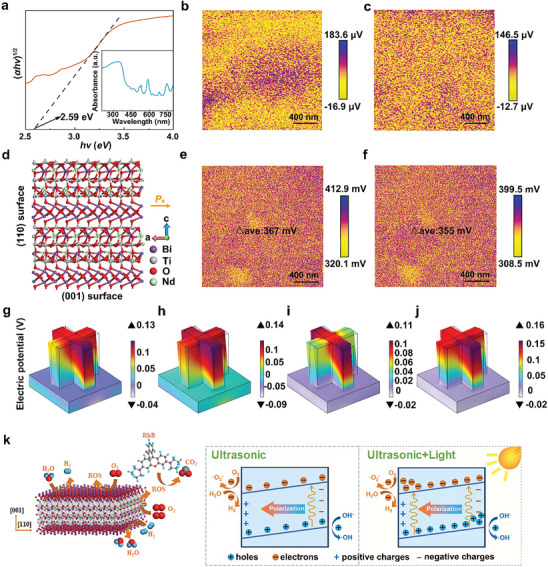
Optical properties, piezoelectric properties and theoretical analysis of BITNd‐20 composite ceramics. a) UV–vis diffuse reflectance spectra and the corresponding band gap, b) in‐plane, c) out of plane PFM amplitudes. d) Crystal structure and ferroelectric polarization of BIT‐Nd. KPFM potential images e) in the dark and f) under the illumination of BITNd‐20 composite ceramics. g–j) COMSOL simulations of the piezoelectric potential distribution in BITNd‐20 composite ceramics under 100 MPa pressure for four different polarization arrangements. k) Mechanisms of the catalytic process with the application of ultrasound and a combination of ultrasound and light.

A UV–vis diffuse reflectance spectrum (DRS) and Tauc's plot of the BITNd‐20 composite ceramic are shown in **Figure** **5**a. It can be clearly seen that the BITNd‐20 composite ceramics exhibited strong absorption bands within the wavelength range of 200–800 nm, with the absorption edge at ≈450 nm. By employing the Kubelka‐Munk method with (𝛼ℎ𝑣)^1/2^ as the ordinate and *hv* as the abscissa, the band gap of BITNd‐20 composite ceramic can be obtained as 2.59 eV by analysing the straight‐line portion of the curve and determining the tangent line to intersect with the *x*‐axis. Contact mode piezo‐force microscopy (PFM) was applied to directly characterize the piezoelectricity of BITNd‐20 composite ceramic. Figure 5b, and c show the in‐plane and out‐of‐plane PFM amplitude of BITNd‐20 composite ceramic, respectively. The corresponding phase diagrams showing the polarization direction are shown in Figure S14 (Supporting Information). It was evident from Figure 5b that the contrast in the in‐plane PFM amplitude due to ferroelectric domains was more prominent in comparison to the surrounding nonpolar matrix, reaching a maximum amplitude of 183.6 uV. However, the level of contrast in the out‐of‐plane polarisation component of the PFM amplitude image shown in Figure 5c was weaker, with a maximum amplitude of 146.5 uV. The maximum in‐plane PFM amplitude of BIT‐Nd was ≈1.25 times that of the out‐of‐plane amplitude, suggesting that the in‐plane piezoelectric response of this system was stronger than the out‐of‐plane piezoelectric response. In addition, COMSOL simulations revealed that in the case of a strong in‐plane polarisation, the stress on the exposed surfaces of (110) and (001) leads to a substantial difference in piezoelectric potential output. When a force was applied to the (110) face, the the piezoelectric potential output was 3.81 V, significantly higher than the piezoelectric potential of 0.27 V when the force was applied to the (001) face, as shown in Figure S15 (Supporting Information). Therefore, the exposed (110) face of the nanosheets is the preferred surface for the application of a force to provide a large piezoelectric output. This also explained why the piezocatalytic activity of BITNd‐*x* (*x* = 10, 20, 30) was significantly improved compared to BITNd‐0 composite ceramics. The crystal structure and ferroelectric polarization of BIT‐Nd are shown in Figure 5d.

In addition, the migration characteristics of photo‐generated charges during the catalytic process were investigated. Kelvin Probe Force Microscopy (KPFM) was utilized to record the changes in surface potential of the BITNd‐20 composite ceramic in both dark and illuminated conditions, as shown in Figure 5e,f. In the dark, the average surface potential difference of BITNd‐20 composite ceramic was measured at 367 mV. With illumination, the average surface potential difference of BITNd‐20 composite ceramic decreased to 355 mV. This can be explained by the migration of photo‐generated charges driven by the potential difference to the opposite positions of the catalyst, leading to a reduction in surface potential due to the charge screening effect.^[^ ^39^ ^]^

Finite element modelling (FEM) simulations were used to analyze the difference in piezoelectric potential output due to nanosheets grown on the (110) and (001) faces of the substrate. The model size was established according to Figure 2. According to the principle of minimum energy, when the dipole moments of neighboring domains are aligned at 90°, the total electric moment of the crystal is minimized, resulting in the most stable state of the crystal. The four possible arrangements of the polar axis are illustrated in Figure 5g–j. Models in Figure 5g,h show nanosheets grown along the *a‐b* axis (corresponding to the pre‐growth stage of the nanosheets) on the (110) face of the substrate micron‐scale powder. In Figure 5g, the polarization direction of substrate micron‐scale powder was upward, resulting in a piezoelectric potential output of 0.17 V, while in Figure 5h, with the polarization direction of substrate micron‐scale powder downward, the output was 0.23 V. This contrast in output was attributed to the selection of the zero potential surface. Figure 5i,j show models with nanosheets of the substrate micron‐scale powder grown on the (001) surface (corresponding to the late stage of nanosheet growth). Here, the piezoelectric potential output was influenced by the surface nanosheet polarization direction. Specifically, the upward polarization direction of the surface nanosheets yielded a piezoelectric output of 0.13 V, whereas the downward polarization direction resulted in an output of 0.18 V. Notably, the average piezoelectric potentials of BITNd‐20 composite ceramics in this case were higher than those depicted in Figure 5g,h. Therefore, nanosheets grown along the *a‐b* axis on the micron‐scale powder (110) crystal face of the substrate exhibited a higher piezoelectric potential output, which was more conducive to piezoelectric catalysis.

Figure 5k displays the crystal structure of BITNd‐*x* composite ceramics, where it is a typical layered perovskite structure composed of bismuth oxide and perovskite‐like layers stacked along the [001] direction. Due to its unique 2D layered structure, it has been demonstrated previously to exhibit anisotropy in spontaneous polarization, hydrogen adsorption free energy, and piezoelectric potential output. Consequently, it generates more free radicals and higher HER activity on the (110) surface. As shown in Figure 5k, when the BITNd‐*x* composite ceramics are subjected to ultrasound in the dark state, the ferroelectric ceramics undergo variation in spontaneous polarization due to the application of alternating stress. This disrupts the temporary balance of charges and screened charges, enabling the adsorption and desorption of surface charges, which in turn makes the BITNd‐*x* composite ceramics efficient in dye degradation and hydrogen production. The BITNd‐*x* composite ceramics generate a large number of photogenerated carriers and a small amount of thermally generated carriers. Under the driving force of the built‐in electric field, these carriers are effectively separated and moved to the surface of the material, where they participate in chemical reactions. This significantly enhances degradation performance and hydrogen production capability. In summary, the excellent piezo‐photo catalytic performance of BITNd‐20 composite ceramics can be specifically attributed to several factors: i) the presence of nanosheets and alignment pores can enhance light reflection and accessibility for water flow, improving the utilization efficiency of light waves and ceramic interior; ii) the {110} crystal face has the advantages of strong polarity and strong piezoelectric response; iii) the {110} surface has a lower combination rate during charge carrier migration and its high HER activity promotes free radical generation and catalysis. iv) The built‐in electric field generated under ultrasound can greatly inhibit the combination of photo‐generated carriers.

## 3 Conclusion

The study has developed a new hierarchical design strategy to develop a high‐performance piezo‐photocatalytic Bi_3.15_Nd_0.85_Ti_3_O_12_‐*x* (BIT‐Nd‐*x*) composite ceramic based on the piezoelectric‐photovoltaic effect, effectively addressing the issue of secondary pollution for water caused by powder‐based catalytic materials. Initially, BIT‐Nd micro‐meter scale particles prepared by solid‐state reaction method were used as raw materials, followed by the fabrication of porous BIT‐Nd ceramics with aligned pores using freeze‐casting. Subsequently, the ceramics were surface‐modified with nanosheets through hydrothermal treatment to obtain BITNd‐*x* composite ceramics. Observation of the nanosheet growth process revealed preferential growth on the {110} surface of the micron‐sized powders along the *a‐b* axis, followed by growth within the {001} surface. At this stage, the nanosheets are able to expose the {110} surface, thereby increasing the active area of the {110} surface in BITNd‐*x* composite ceramics. Furthermore, the BITNd‐20 composite ceramic with a porosity of 64.6 vol.% exhibited excellent piezo‐photocatalytic activity, with first order rate constant, *k*, of 0.0321 min^−1^ for Rhodamine B (RhB) degradation and a hydrogen production rate of 1.30 µmol·h^−1^ in pure water. The piezo‐photocatalytic first‐order rate constant, *k*, values of the composite ceramics were observed to be higher than recently reported bulk catalysts. The composites also achieved ≈90% degradation of colorless pollutants at 10 mg L^−1^ within 100 min. A combination of detailed experiments and modelling revealed the advantages of the {110} surface over the {001} surface in piezoelectric response, the free energy of hydrogen adsorption, and piezoelectric potential output, demonstrating the significant enhancement in piezo‐photocatalytic activity. This work, therefore, provides a new approach for the development of future piezoelectric ceramics with high piezo‐photocatalytic activity and provides significant potential for piezo‐photocatalytic applications related to hydrogen production and water treatment.

## 4 Experimental Section

### Chemical and Reagents

Analytical grade Bi_2_O_3_ (99.9%), Nd_2_O_3_ (99.99%), TiO_2_ (99.0%), Bi(NO_3_)_3_·5H_2_O (≥99.0%), Nd(NO_3_)_3_·6H_2_O (99.0%), NaOH(≥98%) powders and Tetrabutyl titanate (99.0%) were used as raw materials.

### Preparation of Bi_3.15_Nd_0.85_Ti_3_O_12_ (BIT‐Nd) Powders

BIT‐Nd powders were prepared by solid‐state reaction method. Bi_2_O_3_, Nd_2_O_3_ and TiO_2_ powders were mixed according to the required stoichiometric ratios. The mixed powders were then ball milled with ethanol at 250 r min^−1^ for 24 h. Subsequently, the dried ball‐milled powders were calcined at 800 °C for 2 h and subsequently ball milled for 48 h.

### Preparation of BIT‐Nd Porous Ceramic Matrix

The matrix was prepared by water‐based freeze casting. The total volume of the suspension was 20 mL, and the corresponding mass of BIT‐Nd powders was weighed according to the volume fractions in terms of 25, 30, 35, and 40 vol.%. This mixture was then ball‐milled together with polyacrylates and deionized water for 12 h at 100 r min^−1^. Then, polyvinyl alcohol was added to the suspension as a binder and ball‐milled for 2h. The suspension was transferred to molds and frozen in a liquid nitrogen container with a temperature gradient. The frozen samples were subsequently freeze‐dried under vacuum conditions for 48 h at −25 °C and 1 Pa to sublimate the ice crystals. Finally, the obtained porous green bodies were sintered at 1000 °C for 2 h.

### Preparation of BIT‐Nd Porous Composite Ceramic

BIT‐Nd (Bi_3.15_Nd_0.85_Ti_3_O_12_‐*x*, *x* = 0, 10, 20, 30 wt.%) porous composite ceramics were synthesized by a hydrothermal method. For example, in the case of BITNd‐30, 2.55 g of Bi(NO_3_)_3_ 5H_2_O and 0.621 g of Nd(NO_3_)_3_ 6H_2_O were added to 6 mL of tetrabutyl titanate. Subsequently, a 70 mL NaOH solution with a concentration of 3 M was added to the precursor solution prepared above, followed by magnetic stirring for 0.5 h. The resulting mixture, along with the porous ceramic matrix prepared as described in Section 2.2, was transferred to a 100 mL teflon‐lined stainless autoclave and heated at 200 °C for 24 h. After cooling, the BITNd‐30 composite ceramics were washed with deionized water and ethanol at least three times and then dried in air at 70 °C for 24 h to obtain the BITNd‐30 composite ceramic. BITNd‐20 and BITNd‐10 composite ceramics were synthesized with 1/2 or 1/3 of the preceding mass of Bi(NO_3_)_3_ 5H_2_O and Nd(NO_3_)_3_ 6H_2_O under the same conditions. The *x* values (10, 20, 30 wt.%) represent the mass ratio between the hydrothermal product and the porous composite ceramic. The specific experimental data is shown in Table S1 (Supporting Information).

### Characterization

The phase structure of the samples was analyzed by X‐ray diffraction (XRD, Rigaku SmartLab, Japan). The nanostructure of the samples was characterized using scanning electron microscopy (SEM, TESCAN MIRA3, Czech Republic). Energy‐dispersive Spectroscopy (EDS) was used to analyze the elemental distribution of the materials. X‐ray photoelectron spectroscopy (XPS, Thermo Fisher, USA) was used to qualitatively analyze the elemental composition and valence analysis of the surfaces. The relative permittivity, conductance, phase angle, and dielectric loss were measured at frequencies from 1 to 10^4^ kHz using a precision impedance analyzer (4294A, Agilent Technologies, USA). A ferroelectric analyzer (TF analyzer 2000 E, aixACCT systems, Germany) was used to test the hysteresis loops of the samples with an applied electric field ranging from 50–105 kV cm^−1^. The piezo‐responses of samples were evaluated using a piezo‐response force microscope (NanoMan VS, Veeco, USA).

### Catalytic Performance Evaluation for H_2_ Evolution

The piezocatalytic, photocatalytic, and piezo‐photocatalytic activities of the catalysts were evaluated based on the rate of H_2_ evolution. In which an ultrasonic source (45/80/100 kHz, KQ‐200VDE, China) and a 300 W xenon lamp (PLS‐SXE300E, Perfect light, China) were used to provide periodic mechanical force and simulated solar light source, respectively. A certain amount of catalyst was added to 70 mL of deionized water. Following the completion of the catalytic reaction, the amount of H_2_ production was determined using gas chromatography (Fanwei GC‐6600, China) with a thermal conductivity detector, using high purity N_2_ (99.9995 vol%) as carrier gas.

### Catalytic Performance Evaluation for Dye Degradation

The photocatalytic, piezocatalytic and piezo‐photocatalytic activities of the catalysts were evaluated by degradation experiments on Rhodamine B (RhB), Methyl Orange (MO), and Methylene Blue (MB) dye solutions. A 200 W ultrasonic source (45/80/100 kHz, KQ‐200VDE, China) and a 300 W xenon lamp (PLS‐SXE300E, Perfect light, China) were used to provide periodic mechanical force and solar light source, respectively. A mass of 2.566 g of catalyst was dispersed into 100 mL of RhB dye solution (*C*_0_ = 10, 20, 30 mg L^−1^) to establish the adsorption‐desorption equilibrium between the dye and catalyst in the dark. In the catalytic process, 6 mL of the mixed solution was collected at certain time intervals and subsequently centrifuged at 9500 rpm for 10 min to obtain the supernatant. The changes in dye absorbance were analyzed using a UV spectrophotometer.

For comparison, the degradation rate (D%) and the first‐order kinetic rate constant (*k*) were calculated as Equations (8) and (9).

(8) $$D\%=\left[1-\left(C_{t}/C_{0}\right)\right]\times 100\%$$

(9) $$\ln\left(C_{0}/C_{t}\right)=\mathrm{kt}$$

where *t* is the reaction time, *C_t_* and *C*_0_ are the instantaneous and the original dye concentration, respectively, and *k* can be obtained from the ln (*C*_0_ *C_t_)* – *t* plot.

### Finite Element Simulation

A 3D model was built using NX 12 and then imported into COMSOL Multiphysics software. By setting the material type, mesh division, polarization direction, fixed constraints and grounding plane, the software enables the simulation of potential distribution, electric field distribution, and deformation of the model under various boundary loads applied to the catalyst.

### Density Functional Theory (DFT) Calculations

First‐principles calculations were implemented using Materials Studio. Generalized Gradient Approximation (GGA) in the form of Revised Perdew‐Burke‐Ernzerhof (PBE) functional and OTFG ultrasoft pseudo‐potentials were employed for the DFT exchange correlation energy and the core electrons. A plane‐wave basis set with an energy cut‐off of 489.8 eV and a vacuum layer of 15 Å was established along the Z‐axis to prevent periodic interactions. The relevant formulas are listed below.

i) Δ*E_ads_* is calculated based on the energies of the BIT‐Nd surface with hydrogen adsorbed, clean BIT‐Nd surface, and gas phase hydrogen molecule.

(10) $$\Delta \;E_{ads}=E\left(BITNd+H\right)-E\left(BITNd\right)-\frac{1}{2}E\left(H_{2}\right)$$

ii) The zero‐point energy of the pure surface can be neglected, ΔZPE was calculated by

(11) $$\Delta \;E_{ZPE}=E_{zpe}^{H}\;-\frac{1}{2}E_{zpe}^{H_{2}}$$

iii) As the phonon mode changes of the adsorbed H* on the surface can be neglected, the entropy change can be corrected as follows:

(12) $$\Delta S=-\frac{1}{2}S_{H_{2}}^{0}$$

iv) The pH correction to describe the pH effect from solvation was calculated by

(13) $$\Delta G\;\left(pH\right)=\;-kT\times \ln\left(10\times pH\right)$$

## Conflict of Interest

The authors declare no conflict of interest.

## Supporting information



Supporting Information

## Data Availability

The data that support the findings of this study are available from the corresponding author upon reasonable request.
