# Supplementary material for: {110} Surface‐Exposed Bi3.15Nd0.85Ti3O12 Ferroelectric Nanosheet Arrays on Porous Ceramics as Efficient and Recyclable Piezo‐Photocatalysts
Source: Small. 2025 Jan 27;21(9):2410145. doi: 10.1002/smll.202410145 (PMC11937749; doi:10.1002/smll.202410145)
Supplement: Supplementary file 1 — Supporting Information [file SMLL-21-2410145-s001.docx]

Supporting Information

{110} Surface-Exposed Bi_3.15_Nd_0.85_Ti_3_O_12_ Ferroelectric Nanosheet Arrays on Porous Ceramics as Efficient and Recyclable Piezo-Photocatalysts

Yan Zhao^1^, Yan Zhang^1,^*, Xuefan Zhou^1,^*, Kaiyu Feng^1^, Qianqian Xu^1^, Hanyu Gong^1^, Di Zhai^1^, Mingyang Yan^1^, Dou Zhang^1^, Chris Bowen^2^

1 State Key Laboratory of Powder Metallurgy, Central South University, Changsha, Hunan 410000, China

2 Department of Mechanical Engineering, University of Bath, Bath, BA2 7AY, UK

E-mail: [yanzhangcsu@csu.edu.cn](mailto:yanzhangcsu@csu.edu.cn); [zhouxuefan@csu.edu.cn](mailto:zhouxuefan@csu.edu.cn)


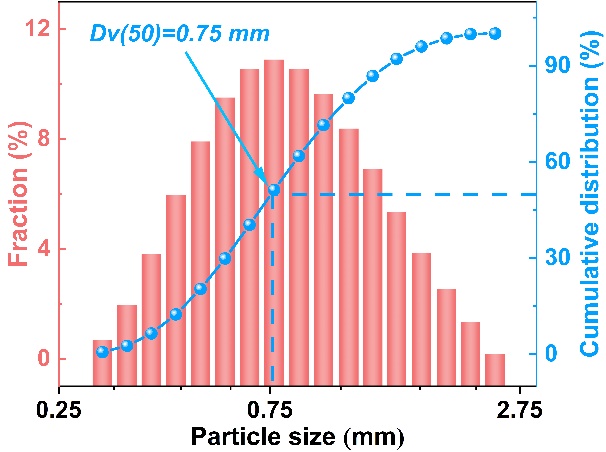


**Figure S1.** Particle size distribution of the BIT-Nd powders.


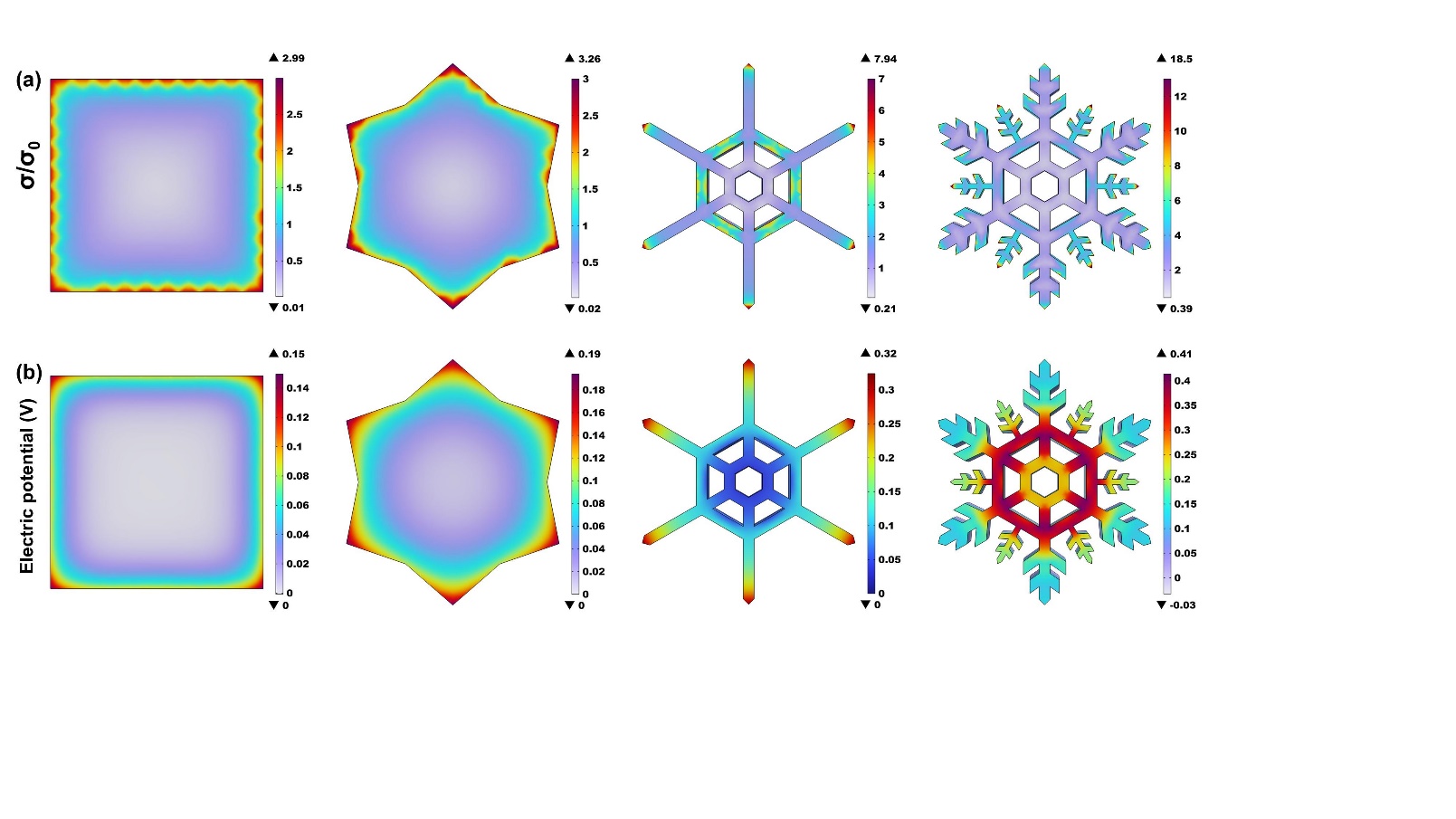


**Figure S2.** Stress distribution and piezoelectric potentials of different shapes simulated by COMSOL.


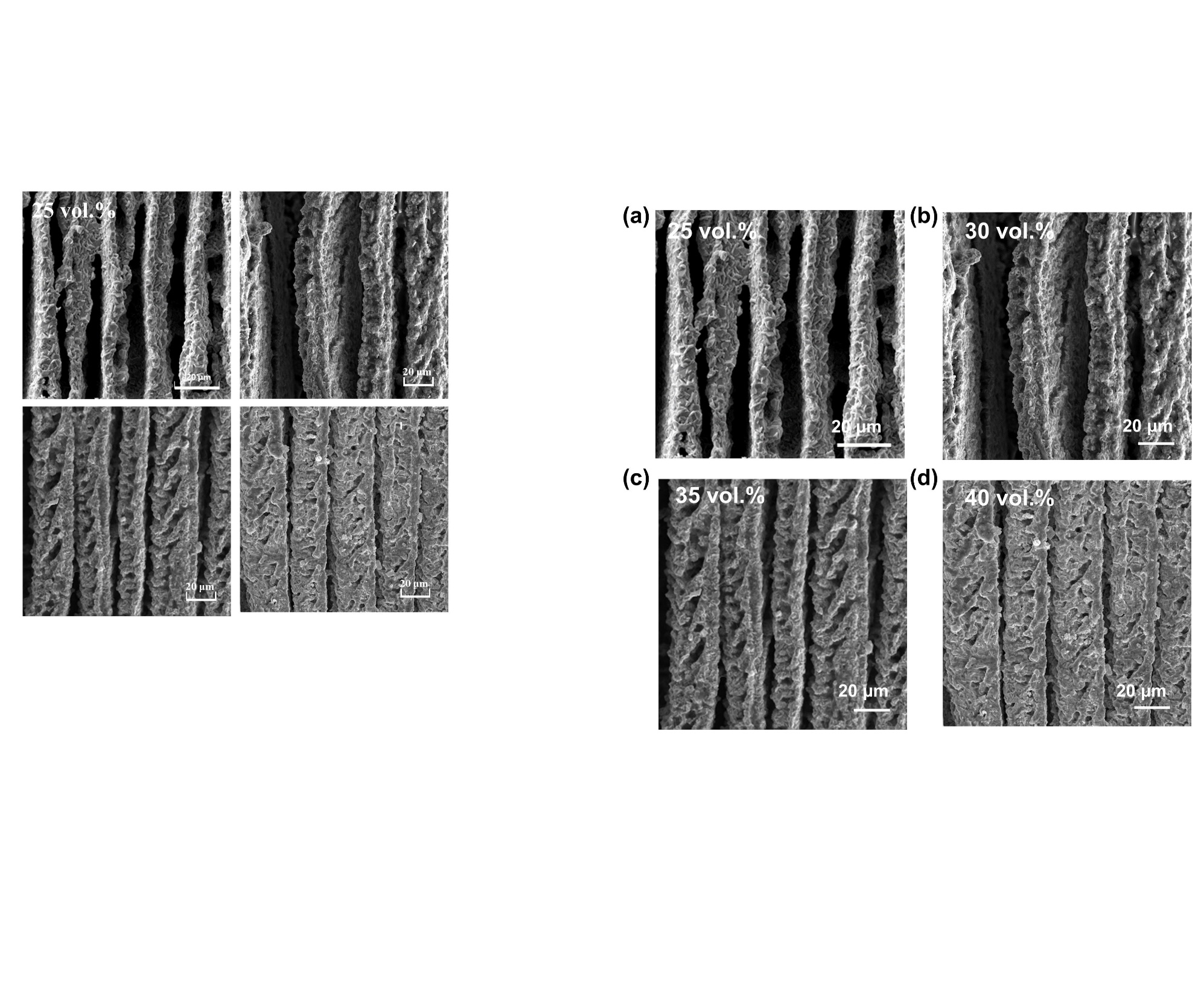


**Figure S3.** SEM images of BIT-Nd aligned porous ceramics with solid loadings of (a) 25 vol%, (b) 30 vol%, (c) 35 vol% and (d) 40 vol%


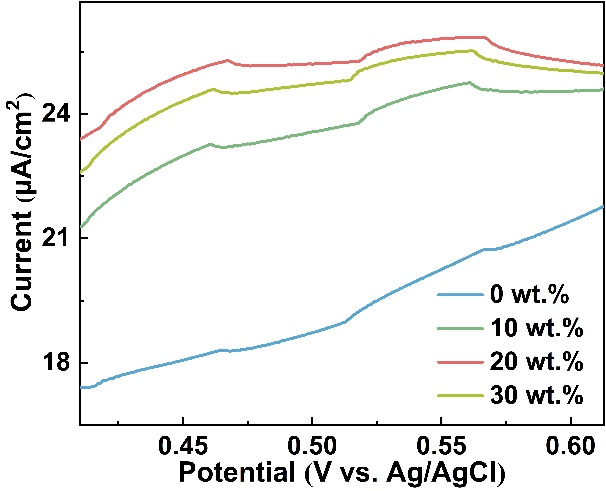


**Figure S4.** Photocurrent response of BITNd-*x* composite ceramics.


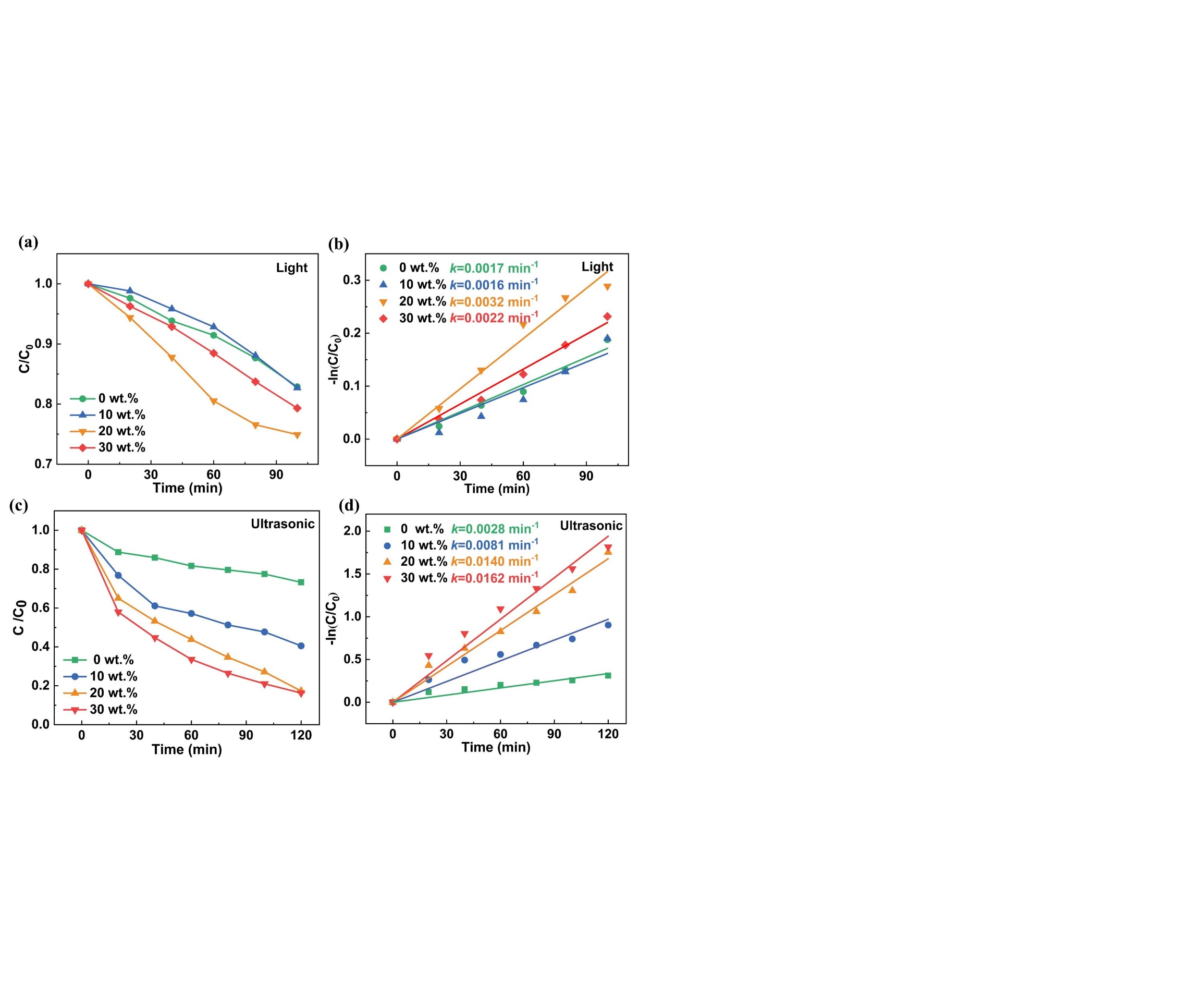


**Figure S5.** Piezoelectric catalytic degradation of RhB (*C_0_* = 10 mg/L) by BITNd-*x* composite ceramics (a) concentration variation-time curves and (b) first-order kinetic rate constant.


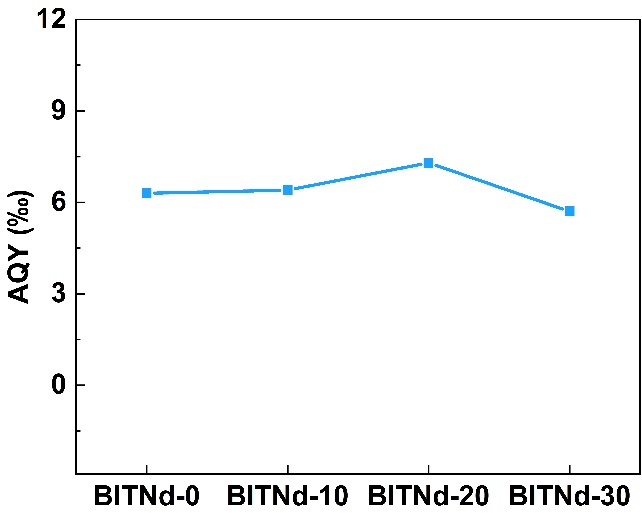


**Figure S6.** Apparent Quantum Yield (AQY) of different samples. Reaction conditions: 2 g catalyst; 70 mL pure water; 350 nm laser (10 W) for 4 h.


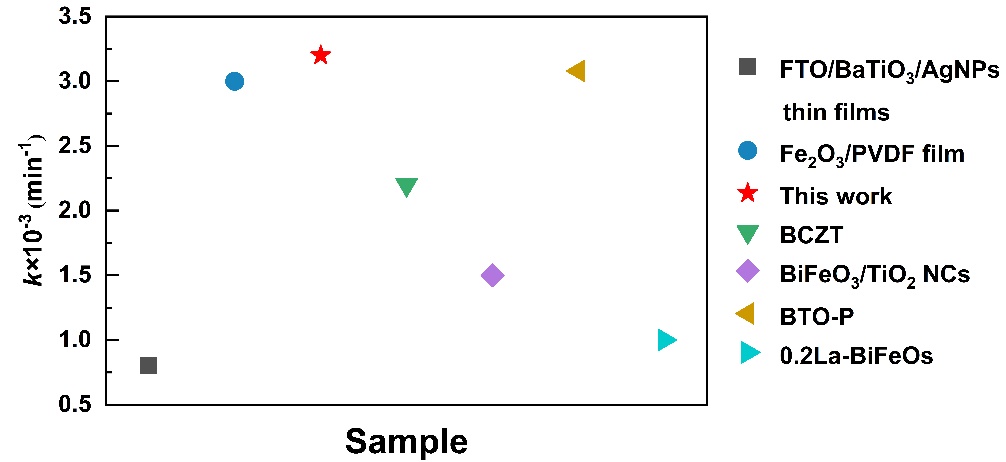


**Figure S7.** The comparison of the photocatalytic performance of BITNd-20 with related materials reported in the literature.


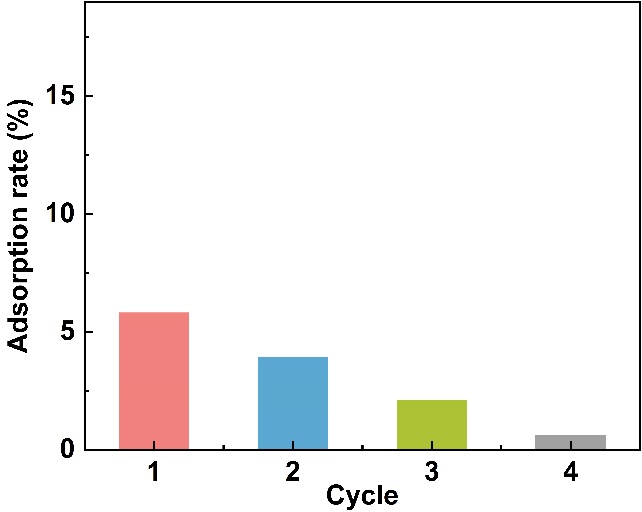


**Figure S8.** Adsorption rate of BITNd-20 composite ceramics during cyclic testing.


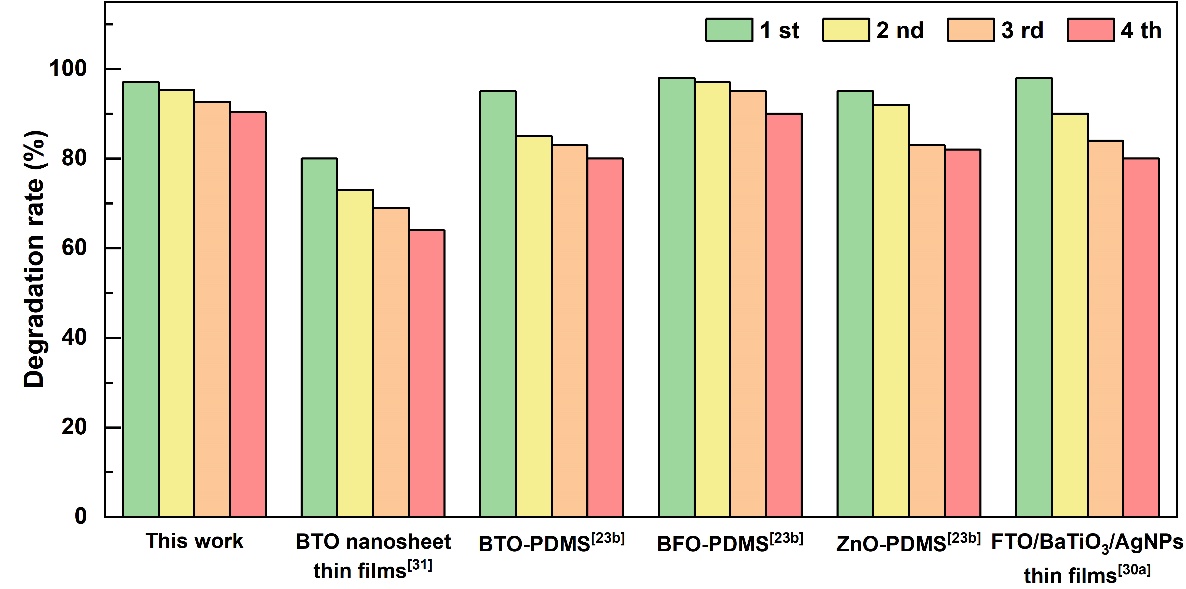


**Figure S9.** Comparison of cycling stability with previously reported work.


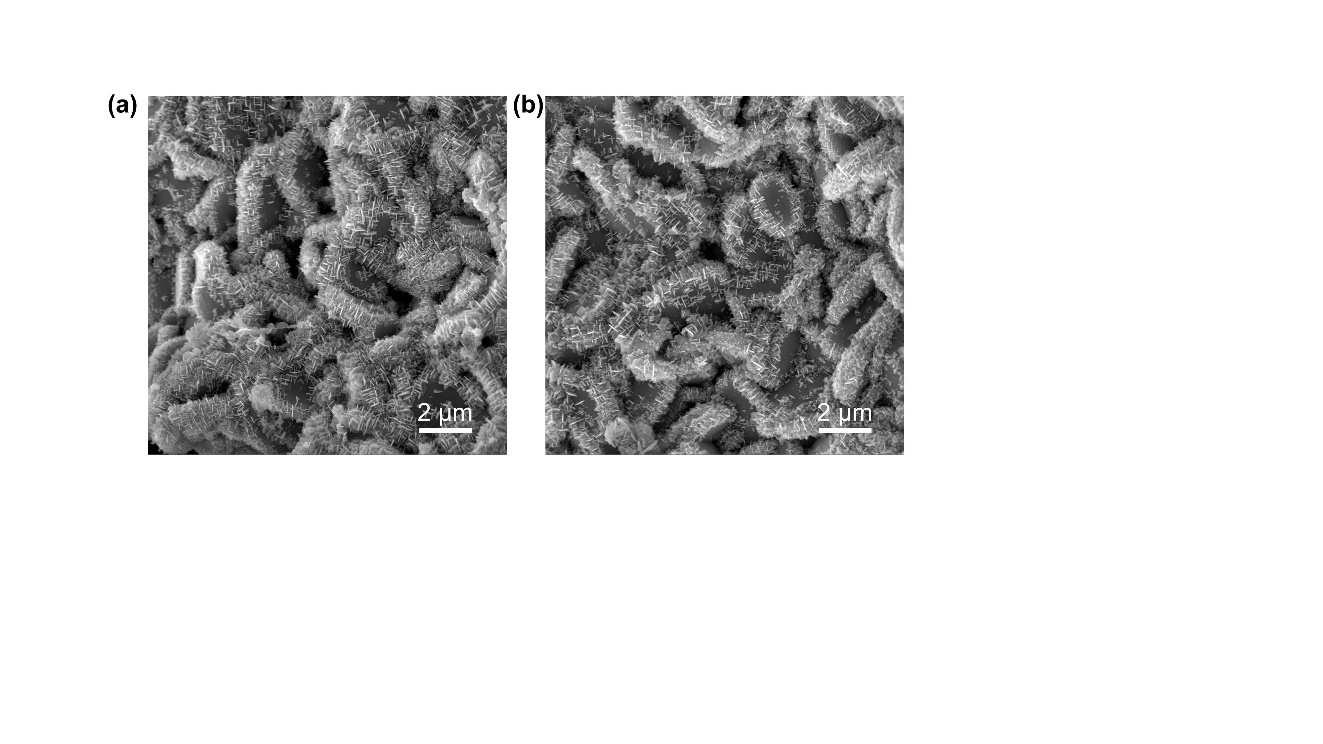


**Figure S10**. SEM images of BITNd-20 before (a) and after (b) the catalytic process.
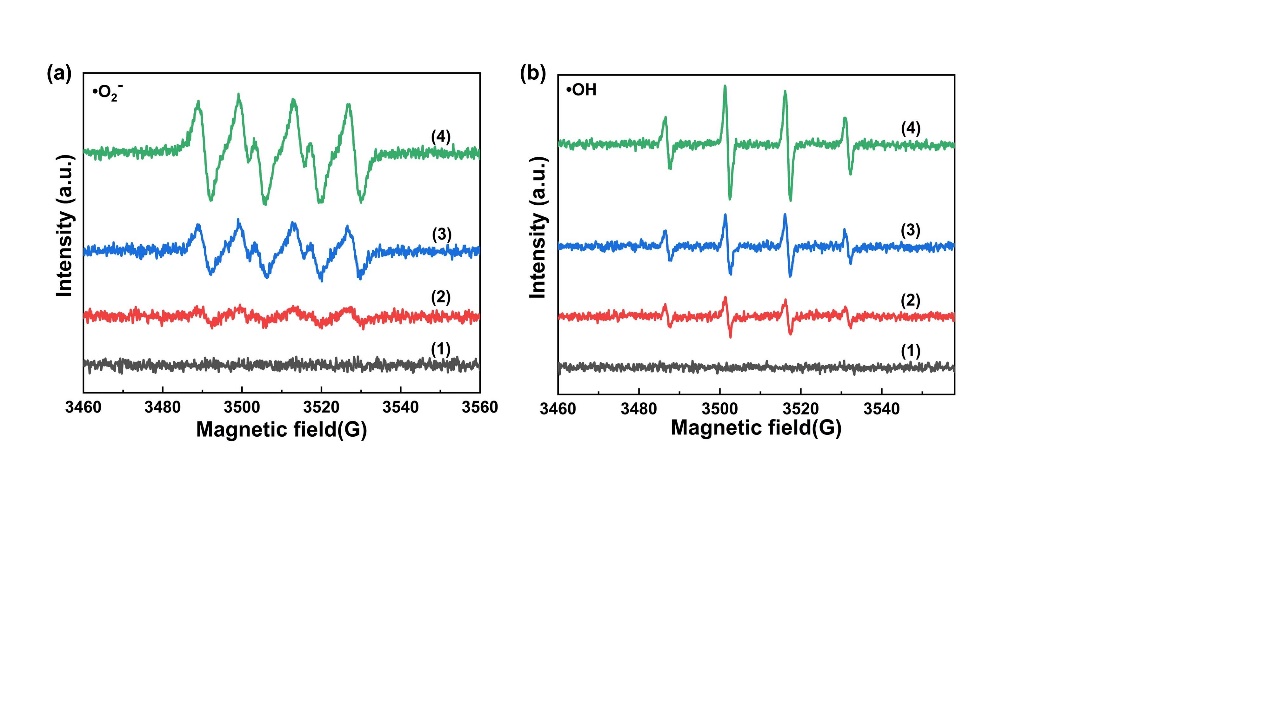


**Figure S11.** EPR measurements of (a) •O_2_^−^ and (b) •OH with radical spin-trapped by DMPO over (1) Pure water+ Light + Ultrasound; (2) BITNd-20 + Ultrasound; (3) BITNd-20 + Light and (4)BITNd-20 + Light + Ultrasound, respectively.

.


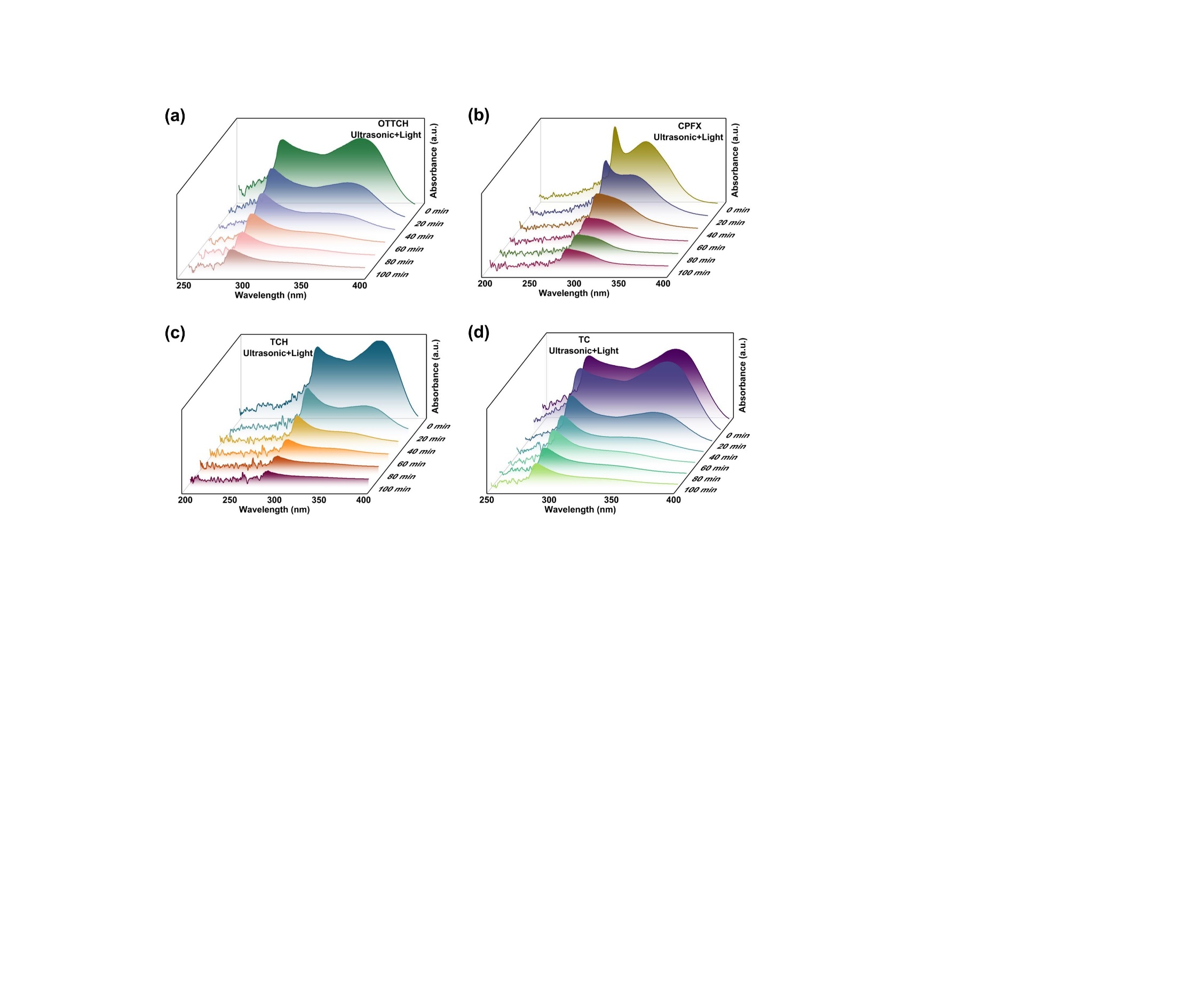


**Figure S12.** UV-Vis absorption spectra of BITNd-20 composite ceramics for piezo-photocatalytic degradation of colorless pollutants over time: (a) OTTCH, (b) CPFX, (c) TCH, and (d) TC.


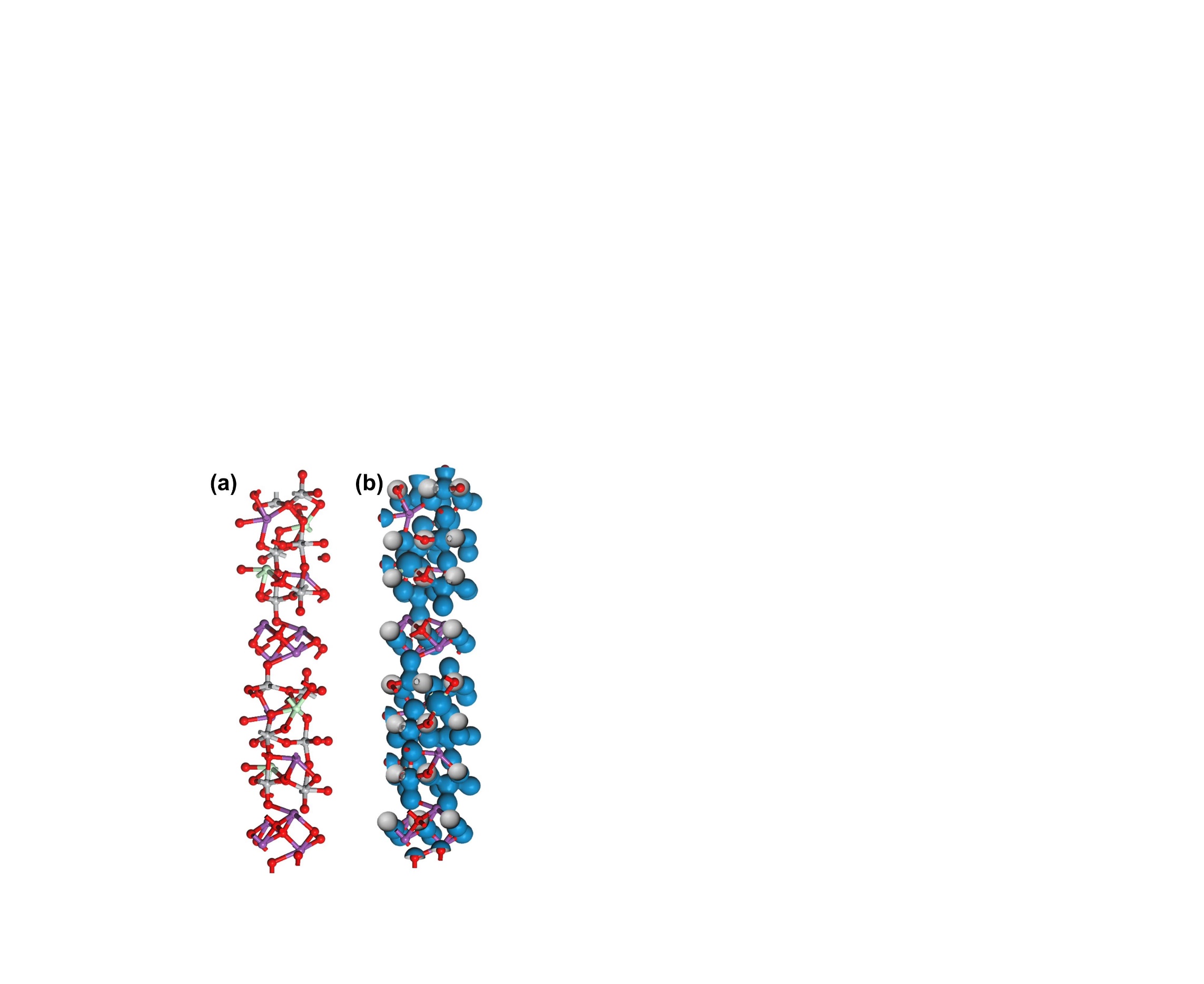


**Figure S13.** Atomic structure diagrams and isosurface total electron density distribution of atoms of (001) crystal planes in BIT-Nd nanosheets (the atoms in red, purple, gray and cyan represent O, Bi, Ti and Nd, respectively. The blue areas represent the total electron density distribution at the isosurface value of 0.5 eÅ^−3^).


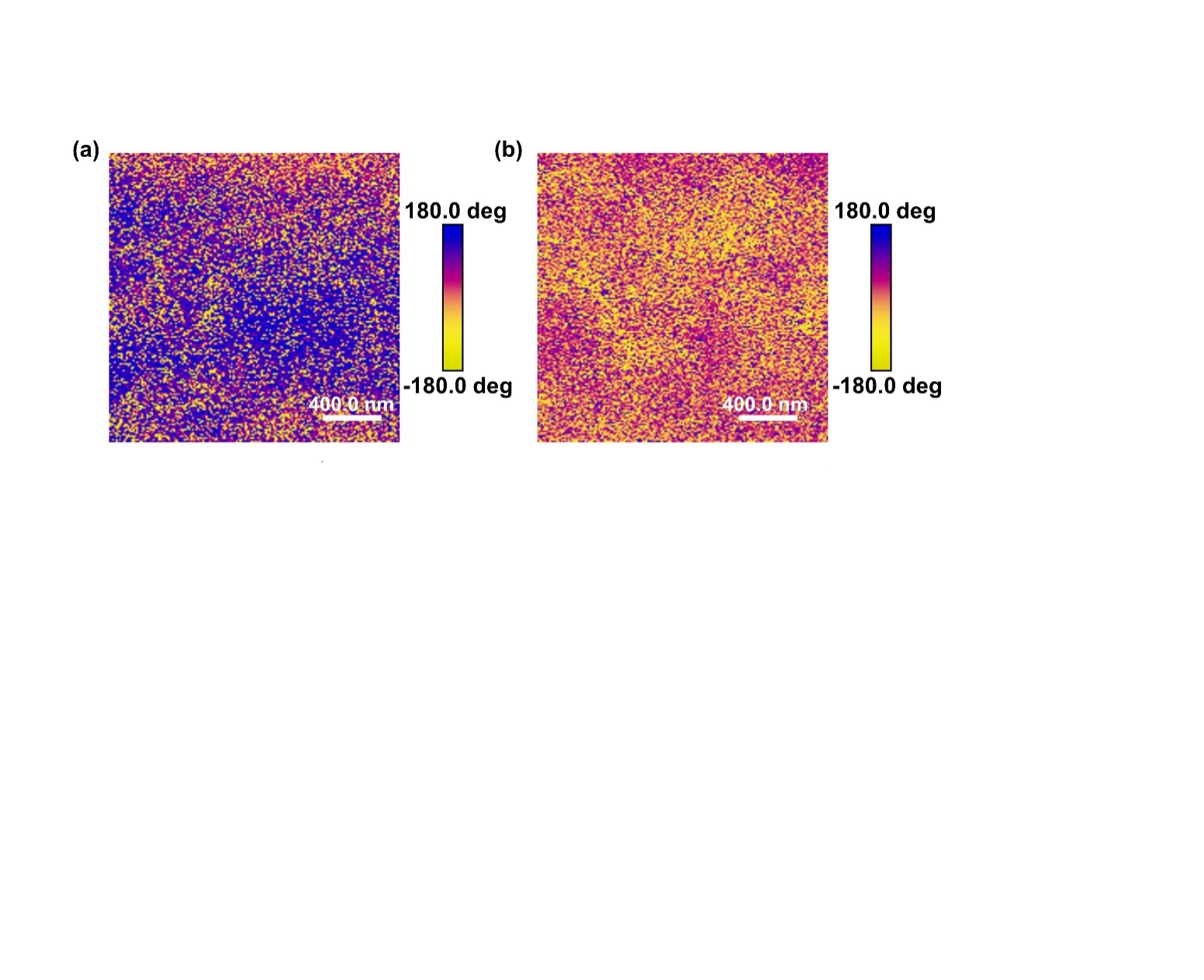


**Figure S14.** (a) In-plane and (b)out of plane PFM phases of BITNd-20 porous composite ceramics.


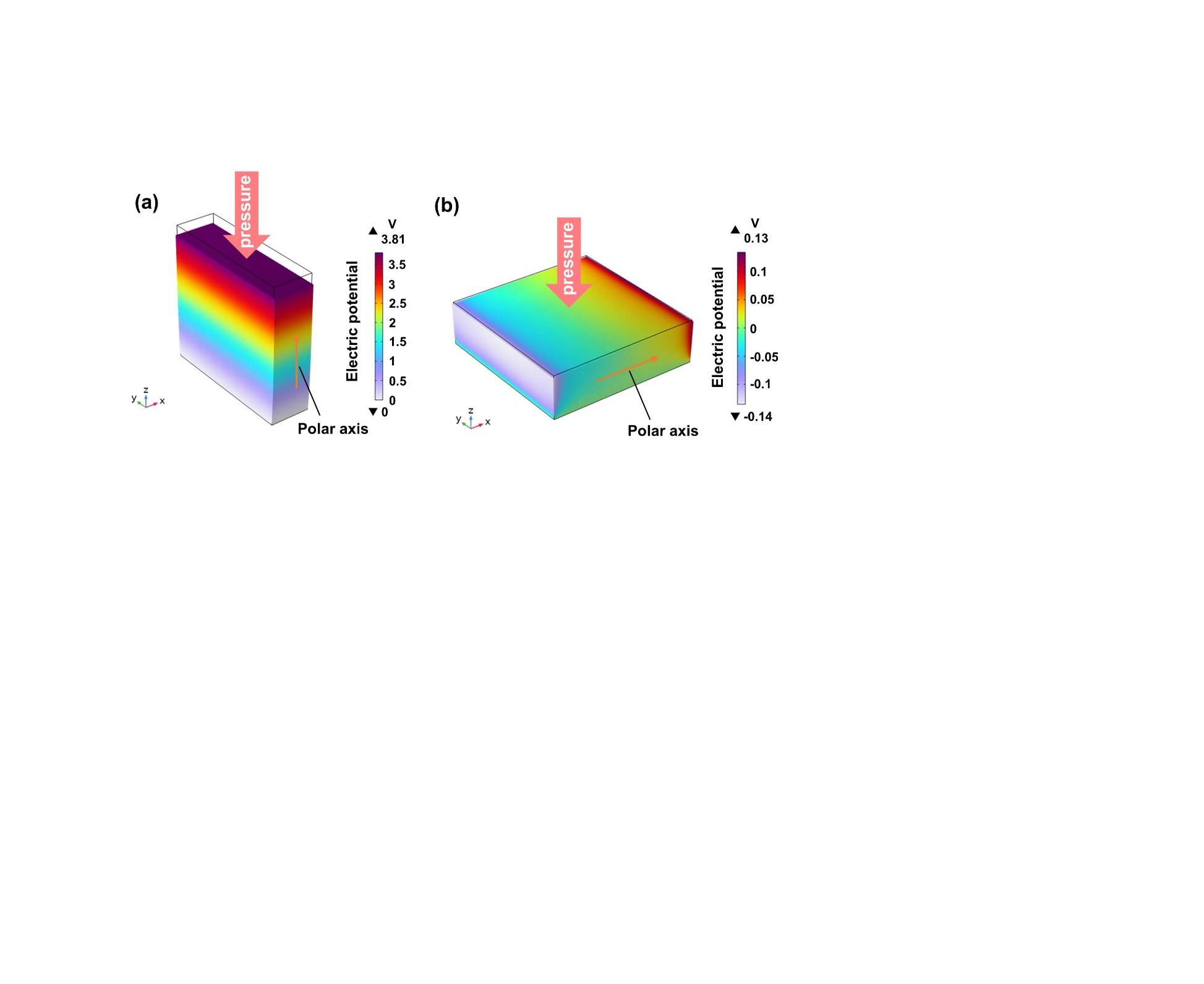


**Figure S15.** COMSOL simulation of piezoelectric potential output of nanosheets when exposed surfaces (a) (110) and (b) (001), where pressure the is 100 MPa.

**Table S1.** The specific qualities of the ceramics.

| Catalyst | A | B | $Ratio=\frac{B}{A+B}$ | *C* |
| --- | --- | --- | --- | --- |
| BITNd-0 | 4.00 | 0 | 0 | 0 |
| BITNd-10 | 4.00 | 0.53 | 0.1 | 0.34% |
| BITNd-20 | 4.00 | 1.05 | 0.2 | 0.48% |
| BITNd-30 | 4.00 | 1.58 | 0.3 | 0.98% |

A= $Mass of the porous ceramic matrix (g)$

B= $Mass of the hydrothermal method product (g)$

C=$\frac{Mass of the composite ceramic-Massof the porous ceramic matrix}{Mass of the porous composite ceramic}$
